# Supplementary material for: Antioxidants N-Acetylcysteine and Vitamin C Improve T Cell Commitment to Memory and Long-Term Maintenance of Immunological Memory in Old Mice
Source: Antioxidants (Basel). 2020 Nov 19;9(11):1152. doi: 10.3390/antiox9111152 (PMC7699597; doi:10.3390/antiox9111152)
Supplement: Supplementary file 1 [file antioxidants-09-01152-s001.pdf]

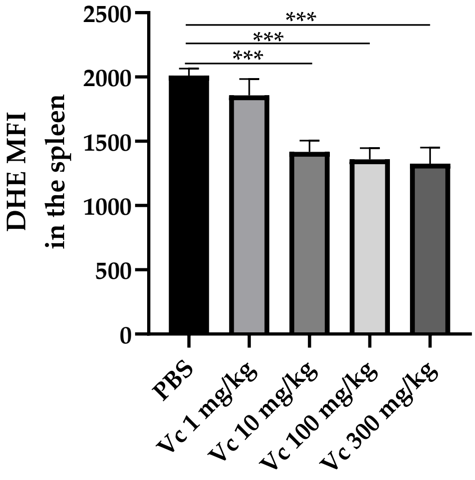

Figure S1. Titration of optimal Vc concentration to use in the study. 1 mg/kg, 10 mg/kg, 100 mg/kg and 300 mg/kg Vc were i.p. injected into the mice from day -7 until day 21, as reported in Figure 1. Alum-OVA was administered on days 0, 14 and 77 and spleen was harvested on day 91. n=3 in each group. DHE MFI (ROS levels) are reported in the Figure. Data are shown as mean ± SEM. One-way ANOVA, Tuckey post-hoc test. ***$p < 0.001$.

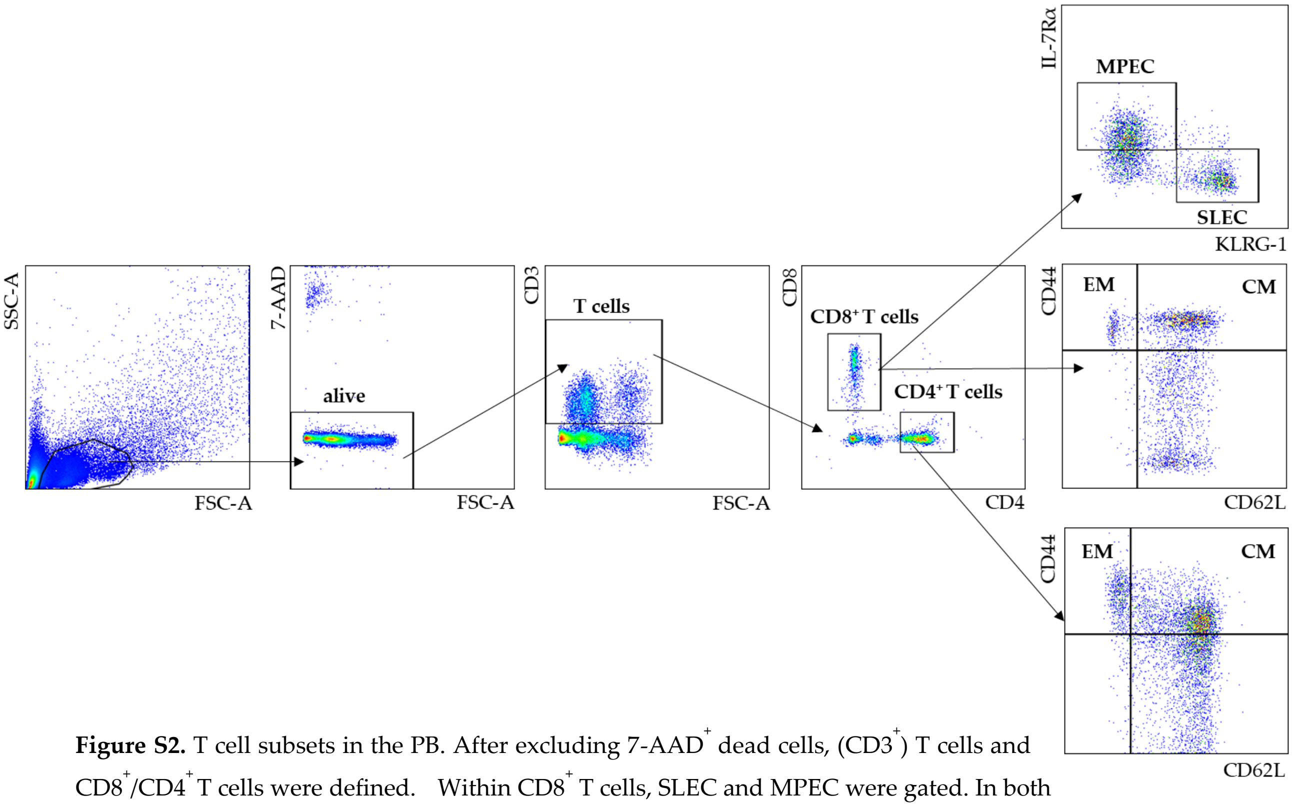

Figure S2. T cell subsets in the PB. After excluding 7-AAD^{+} dead cells, (CD3^{+}) T cells and CD8^{+}/CD4^{+} T cells were defined. Within CD8^{+} T cells, SLEC and MPEC were gated. In both CD8^{+} and CD4^{+} T cells, EM and CM cells were defined.

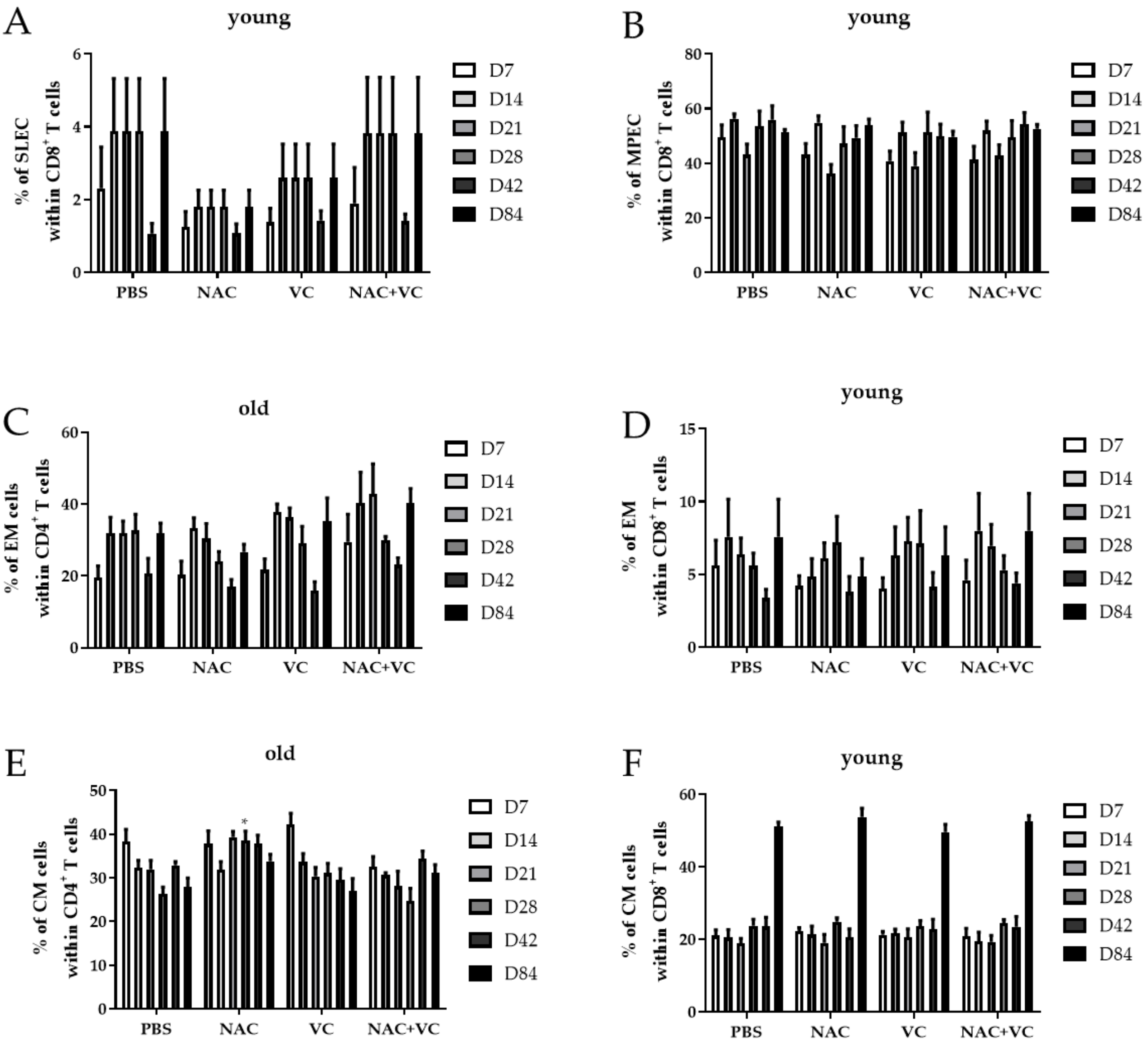

Figure S3. Effector/memory T cell subsets in the PB of young and old mice. Frequency of (A) SLEC in young mice, (B) MPEC in young mice, (C) EM $CD4^+$ T cells in old mice, (D) EM $CD8^+$ T cells in young mice, (E) CM $CD4^+$ T cells in old mice and (F) CM $CD8^+$ T cells in old mice treated with PBS, NAC, Vc or NAC+Vc. Blood was harvested on days 7, 14, 21, 28, 42 and 84. Data are shown as mean ± SEM. Two-way ANOVA, Tukey post-hoc test. $*p < 0.05$.

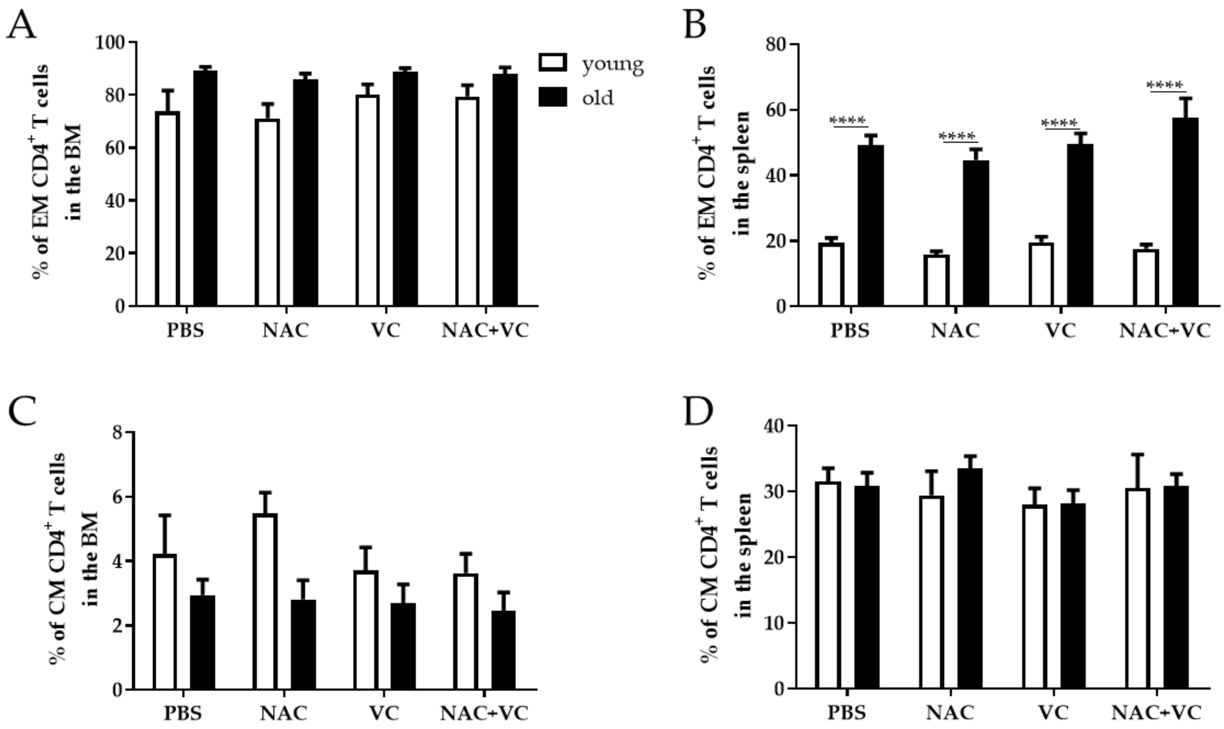

Figure S4. Effector/memory T cell subsets in the BM and spleen of young and old mice. Frequency of (A) EM $CD4^+$ T cells in the BM, (B) EM $CD4^+$ T cells in the spleen, (C) CM $CD4^+$ T cells in the BM, and (D) CM $CD4^+$ T in the spleen of young (white columns) and old (black columns) mice treated with PBS, NAC, Vc or NAC+Vc. Data are shown as mean ± SEM. Two-way ANOVA, Tukey post-hoc test. $^*p < 0.05$.

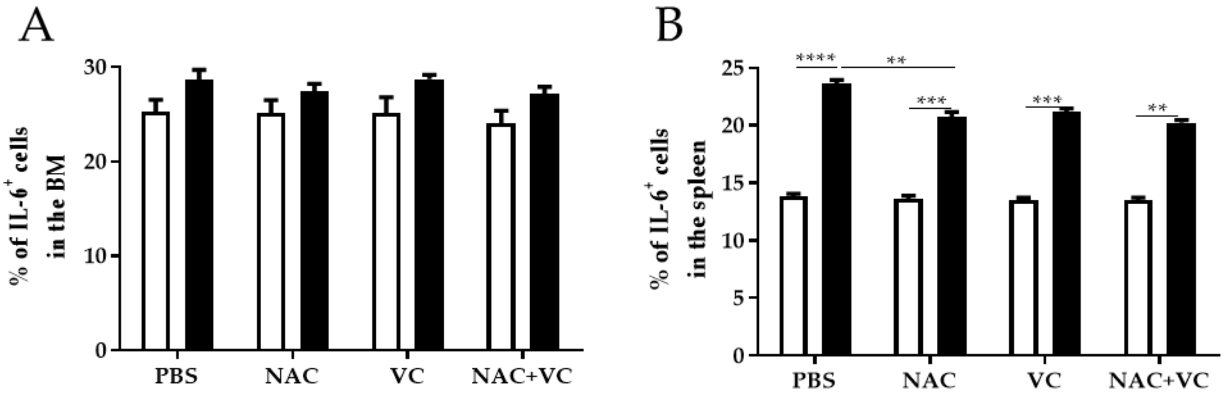

Figure S5. Frequency of IL-6$^+$ cells in (A) BM and (B) spleen of young (white columns) and old (black columns) mice treated with PBS, NAC, Vc or NAC+Vc. Data are shown as mean ± SEM. Two-way ANOVA, Tukey post-hoc test. **p < 0.01; ****p < 0.0001.

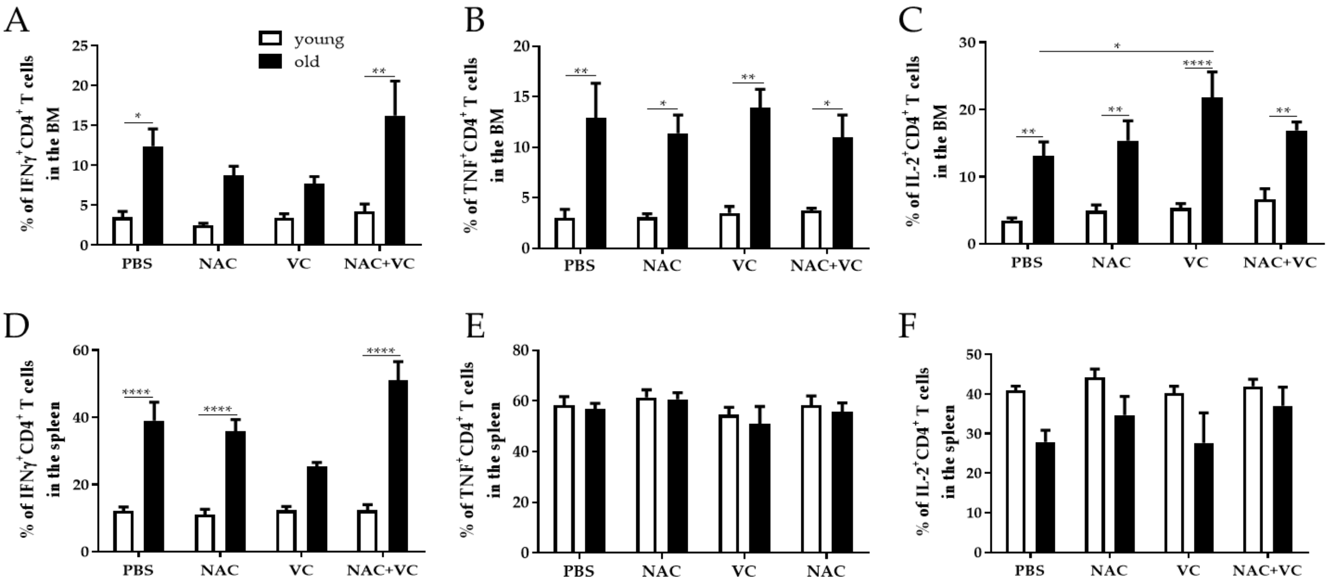

Figure S6. Expression of pro-inflammatory molecules within CD4$^+$ T cells in the BM and spleen. (A) IFNγ$^+$, (B) TNF$^+$ and (C) IL-2$^+$CD4$^+$ T cells in the BM, (D) IFNγ$^+$, (E) TNF$^+$ and (F) IL-2$^+$CD4$^+$ T cells in the spleen of young (white columns) and old (black columns) mice treated with PBS, NAC, Vc or NAC+Vc. Data are shown as mean ± SEM. Two-way ANOVA, Tukey post-hoc test. *$p < 0.05$; **$p < 0.01$; ****$p < 0.0001$.

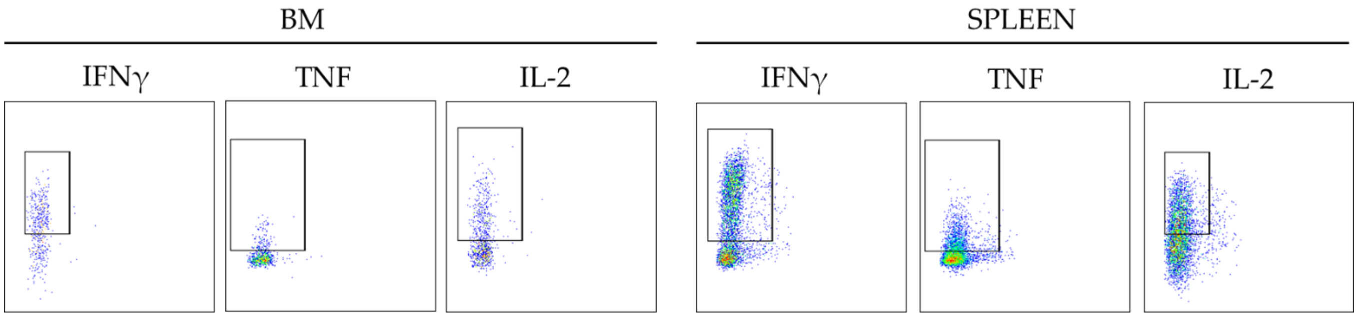

Figure S7. Representative FACS plots showing the expression of IFNγ, TNF and IL-2 within CD8$^+$ T cells from BM and spleen of an old untreated mouse.

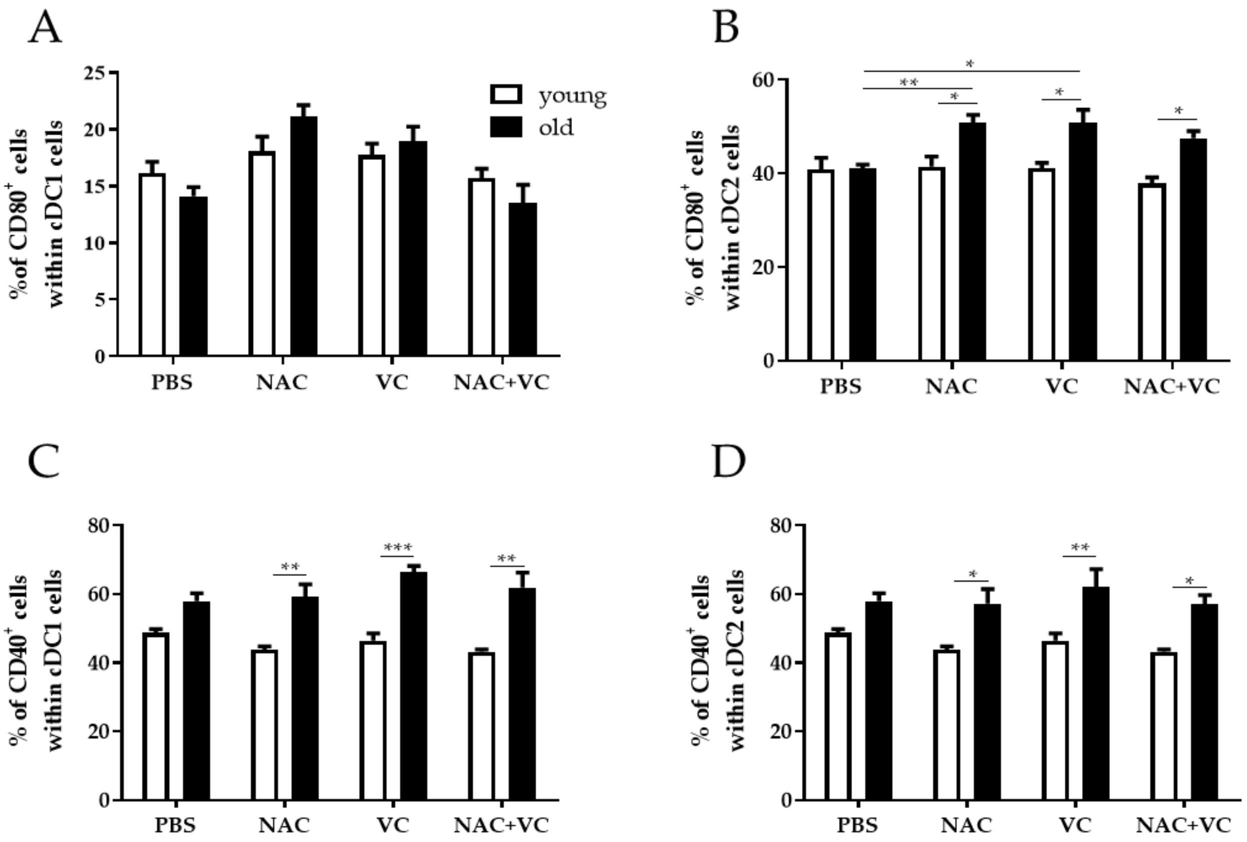

Figure S8. Frequency of CD80 and CD40 in DC subsets. Frequency of (A) $CD80^+$ cells in cDC1 cells, (B) $CD80^+$ cells in cDC2 cells, (C) $CD40^+$ cells in cDC1 cells, (D) $CD40^+$ cells in cDC2 cells in the spleen of young (white columns) and old (black columns) mice treated with PBS, NAC, Vc or NAC+Vc. Data are shown as mean ± SEM. Two-way ANOVA, Tukey post-hoc test. $*p < 0.05$, $**p < 0.01$, $***p<0.001$.
